# Promotion of Student Success and Positive Chemistry Course Perception through Frequent Metacognitive Reporting

**DOI:** 10.1021/acs.jchemed.4c00578

**Published:** 2025-01-01

**Authors:** Michelle Richards-Babb, Carly Gordon, David Mersing, Trina Perrone, Betsy Ratcliff

**Affiliations:** C. Eugene Bennett Department of Chemistry, West Virginia University, Morgantown, West Virginia 26506, United States

**Keywords:** First-Year Undergraduates/General, Philosophy, Assessment, Non-Majors Courses, Student-Centered Learning

## Abstract

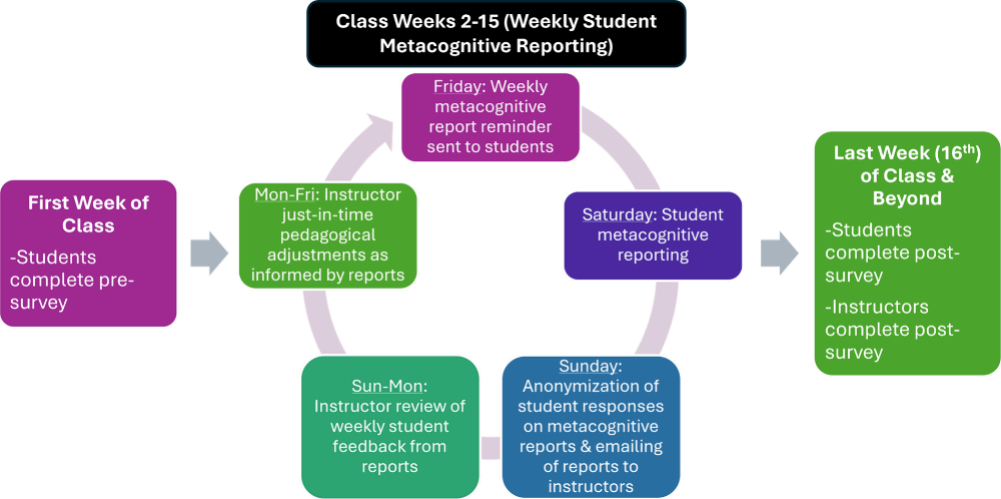

Explicit metacognitive interventions in undergraduate chemistry courses have been shown to improve student outcomes. Less studied have been the outcomes of students who implicitly and frequently practice metacognition and the resultant effects on the student-instructor relationship. In this project set within a large enrollment introductory chemistry course, we elevated student voice and enhanced student-instructor communication through weekly metacognitive reporting to study the characteristics of reporting students and their perceptions of the effects of metacognitive reporting. Data on course success and gender of reporting relative to non-reporting students were quantitatively analyzed using standard statistical techniques. Inductive thematic coding was used to qualitatively assess student responses to open-ended post-survey questions on perceived value of metacognitive reporting. Reporting students finished the semester with a final course grade point average 0.64 points higher than non-reporting students. In addition, reporting students were more successful (more ABC grades) than non-reporting students though small effect sizes and lack of directional causality limit data interpretation. However, female students tended to engage in reporting at a higher rate than did male students. Metacognitive reporting helped to establish a more positive and productive student-instructor relationship via enhanced student communication (i.e., student voice) that informed just-in-time teaching modifications. Students indicated that the metacognitive reporting assisted them in focusing their studies on more challenging topics and in modifying their study habits. In addition, students recognized that their instructors were reading and responding to the reports, which improved overall student-instructor interactions and their view of the instructor as beneficent. Based on these findings, it is recommended that instructors create frequent, low-stakes assignments built into the course structure to support their students’ implicit use of metacognition with the broad goal of growing metacognitive strategies over time.

Educational researchers and instructional practitioners frequently present metacognition as a useful strategy to engage and empower students toward learning.^1,2^ Flavell^3^ gave the first definition of metacognition as “knowledge and cognition about cognitive phenomena” which, over the years, has been generalized to “thinking about your own thinking”. Informing students of the various aspects of metacognition, such as being aware of one’s own thinking and reflecting on one’s experiences, is a tool to improve chemistry learning that has been well-documented in the literature.^4−15^ For instance, Zhao et al. introduced students to metacognitive strategies, including exam performance self-reflection and application of Bloom’s taxonomy, following the first general chemistry exam with frequent semester-long reminders to engage in metacognitive monitoring.^5^ Graham et al. implemented regular, out-of-class, evening peer tutoring sessions that focused on instructing students in metacognitive strategies (e.g., spaced practice, retrieval practice, and interleaving) with concomitant positive impacts on students’ self-efficacy and grades.^4^ Other researchers have addressed:

- metacognition directed at scaffolded problem-solving approaches to specific tasks^7,8^
- explicit teaching of metacognition paired with exam wrappers or reinforced reflective exercises with frequent follow-up reminders to reinforce its use^9,10^
- metacognitive strategies during a semester-long course^11^ or metacognitive regulation during content specific problem-solving^12^
- implicit prediction of quiz score, postdiction of assessments, and the fashioning of study plans^13^
- implicit metacognitive monitoring through postdiction of exam performance^14^
- metacognitive activities as in implicit part of chemistry laboratory experiments involving inquiry,^16^ cooperative problem-based,^17^ and science writing heuristic^18^ pedagogies

In general, metacognitive interventions in first-year chemistry coursework effected positive student outcomes such as improved exam scores,^5,9^ higher final course averages,^4,6^ higher course self-efficacy,^4^ and STEM retention.^11^ Many interventions focused on explicitly informing students about metacognition through lectures or tutoring. When students were asked to practice metacognition associated with lecture courses, it was infrequent and occurred before, after, or during formative or summative assessments. Except for the studies of Casselman and Atwood,^13^ Lopez et al.,^19^ and Pulukuri and Abrams^20^ frequent practice of metacognitive strategies or practice not associated with a lecture exam was limited in the literature.

Flavell further differentiated between cognitive and metacognitive strategies by stating, “Cognitive strategies are invoked to *make* cognitive progress, metacognitive strategies to *monitor* it.”^3^ This definition is particularly apt for the project discussed herein as, on a weekly basis, we prompted students to use metacognitive strategies through reflective writing.^21^ The expectation was that this would correspond to adaptations, allowing for greater success or cognitive progress in learning chemistry. Of note is that students in the present project were not explicitly taught about metacognition or metacognitive strategies. As recommended by others,^9,13^ we provided students with frequent opportunities to practice metacognition because use of metacognition may grow over time. As informed by Lavi et al.^1^ and by Bunce et al.,^22^ the weekly metacognitive prompts used in the present project prompted students to identify challenging topics, motivated students to revisit these topics, and encouraged reflection of chemistry learning.

Parallel to prompting students for implicit use of metacognition, we sought to provide instructors with student-voiced formative feedback to improve student-instructor interactions. In 1987, Chickering and Gamson’s “Seven Principles for Good Practice in Undergraduate Education” publicized the benefits of encouraging “student-faculty contact”.^23,24^ Research indicates the importance of a positive student-instructor relationship. This relationship can affect motivation, commitment toward the course and its content, and ultimately success in STEM.^25−28^ In fact, Christe calls for instructors who are “accountable to students, display positive learner-rapport, and are held to high standards as caring and compassionate educators” (p 24).^25^ Online communication can further strengthen student-instructor contact, especially for students who are less likely to verbalize their questions or concerns in a classroom environment.^29^ Similarly, anonymity in reporting may overcome the student-instructor power dynamics of the classroom.^30^

Student voice, as defined by Seale, is “listening to and valuing the views that students express regarding their learning experiences”.^31^ Many instructors provide informal opportunities for students to voice their opinions, but few formalize these interventions. One method of providing students with a voice concurrent with improving student-instructor contact is through student identification of the “muddiest” point via reflections.^32^ In another study, Gunn used clickers to enhance student-instructor interactions in large-enrollment course sections.^33^ Gunn et al. found that obtaining frequent student feedback informed just-in-time teaching modifications and resulted in high student evaluation scores for instructor openness.

In this project, we elevated student voice via metacognitive reporting to establish a more positive relationship between students and instructors. In addition, we sought to add to the metacognitive literature, specifically focusing on how frequent, implicit metacognitive practice affects student outcomes. Broad goals of this project were to

- provide students with frequent, scaffolded opportunities to implicitly practice metacognition in the expectation of improved student outcomes
- improve student-instructor contact and instructor beneficence (students’ perceptions that instructors truly care about their students)
- provide students with anonymous, student voice on course functioning (content understanding, pedagogy, course structure, and logistics)
- provide instructors with frequent formative feedback from students to enable just-in-time course and teaching modifications for the benefit of all students

Our project informs practitioners, especially instructors responsible for teaching large enrollment chemistry courses to first-year students, of the benefits of providing frequent (i) metacognitive reporting embedded into the course structure and (ii) opportunities for anonymous feedback from students to instructors. Our project addressed the following research questions (RQ):

1. In general, what are the characteristics (e.g., course success, incoming academic demographics, gender) of metacognitive reporters relative to non-reporters in a first-year chemistry course?
2. What do students perceive as the main effects of metacognitive reporting?
3. From a metacognitive standpoint, what advice would students provide to future students for success in the course?
4. Do instructors review and value the weekly metacognitive reports as formative assessment?

## Methods

### Participants

Participants were students enrolled in a 2-credit Introduction to Chemistry (CHEM 110) course at a large, public institution with very high research activity. This course is preparatory and includes 150 min of lecture per week and no laboratory component. The CHEM 110 course is populated by students who, (i) on the basis of their scores on placement or standardized (ACT or SAT) math exams, need further preparation before enrolling in the general chemistry I course (Fundamentals of Chemistry I) for STEM majors or (ii) want a preview of chemistry content to bolster their confidence in college-level chemistry coursework prior to enrolling in the general chemistry I course. A passing letter grade (C– or better) in the CHEM 110 course allows students to directly enroll in general chemistry I. There were 10 sections (*M* = 63 students per section, *N* = 628) of the CHEM 110 course taught by five different instructors during the fall 2022 semester. Course content, syllabus, in-class and outside of class formative and summative assessments, and grade component percentages were consistent between instructors and sections.

A total of 300 distinct CHEM 110 students (47.8%) submitted one or more weekly metacognitive reports and were classified as reporters for this project. Non-reporters were students who submitted zero weekly metacognitive reports. It was expected that even students who submitted few metacognitive reports would experience continued benefits as their instructors provided weekly reminders of metacognitive reporting, which could prompt them to informally ruminate on their chemistry learning. A total of 247 distinct students (39.3%) submitted the pre-survey, and a total of 123 distinct students (19.6%) completed the post-survey. The five instructors were also study participants. Four of the five CHEM 110 instructors submitted the instructor post-survey.

### Measures

Final letter grades and student-level demographic information for each student enrolled in the CHEM 110 course during the fall of 2022 semester were obtained from our institutional registrar.

The four surveys used (the weekly metacognitive report survey, student pre- and post-surveys, and the instructor post-survey) are included as Supporting Information (SI). The weekly metacognitive report survey was based on the Experience Sampling Method (ESM), a form of systemic phenomenography, used by Ye et al. in their 2015 paper.^34^ Systemic refers to how the collected reports together provide a broad overview of students’ experiences. Phenomenography refers to the self-report nature that provides information on individual students’ experiences. Weekly reports were primarily qualitative, with students self-reporting their hours spent attending class, hours spent on additional study, mastery of content, and descriptions of class and additional study activities. Students also had the opportunity to provide input for the instructor on what was helpful to their learning.

A repeated measures pre- and post-survey design was used to collect student data and included statements on learning strategies,^35^ locus of control, and confidence with 5 Likert scale response choices ranging from strongly disagree to strongly agree.

Unfortunately, the low number of participants who could be matched (i.e., pre- to post-surveys) limited the interpretation of changes in Likert scale responses for these qualitative measures. The student post-survey also included open-ended questions. One open-ended question asked students about the effects of weekly embedded reporting (*Did you think about or do anything differently that you would attribute to the reporting? Did writing your reports affect how you studied for your CHEM 110 or other STEM math and/or science classes?*). A second open-ended question (*If you were to advise a future student for success in the CHEM 110 course, what advice would you give? In hindsight, what did you do that was important to your success and what would you have done differently?*) inquired about current student advice to future students for success in the CHEM 110 course. The qualitative instructor post-survey included questions on estimation of percentage of reports read, instructional adjustments implemented, and usefulness of reports beyond instructional adjustments. Validated Likert items pertaining to incentives for instructional innovation were obtained from the literature.^36^

### Design

All five instructors participated in this project and encouraged their students to complete the pre- and post-surveys and 14 weekly metacognitive reports (16 total submission opportunities). Qualtrics was used as the electronic delivery platform for the surveys and reports. As a group, the five instructors agreed to award their CHEM 110 students 0.375 “extra” points for each survey or report submitted. The points were added as “extra credit” to each student’s earned final exam score, which was worth 100 points and 25% of the final numerical grade. Students who took advantage of all 16 submission opportunities could increase their final exam grade by 6 points (16 submissions × 0.375 pts/submission) and their final numerical grade by a maximum of 1.5 percentage points (16 submissions × 0.375 pts/submission × 0.25).

**Figure 1:**
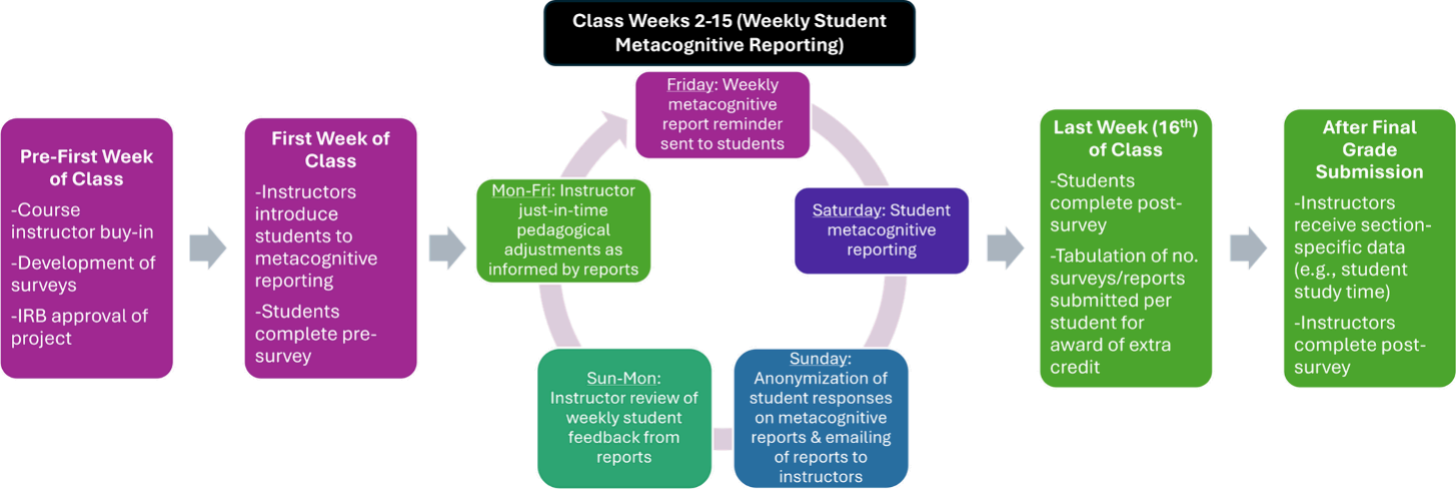
Process diagram of the project’s design including a timeline of major components and when surveys (student pre-/post- and instructor post-) and weekly metacognitive reporting by students were completed.

Overall, a total of 348 distinct students submitted one or more surveys or reports for this project with a mean of 5.4 surveys and reports submitted, resulting in a mean increase in their final numerical grade of 0.51 percentage points (5.4 submissions × 0.375 pts/submission × 0.25). Student pre- and post-surveys were distributed in the first and last (16th) weeks of the semester, respectively. Weekly reporting began in the second week and was offered to students each week until the 15th week. Weekly reports were due by 11:59 PM each Saturday. On the following day, reports were anonymized and sent to instructors so that they could make just-in-time teaching adjustments for the upcoming week. A diagram of the project’s design is provided in Figure 1.

During the semester, instructors were not aware of the identities of students who completed surveys and reports. The project lead was not involved in the administration or teaching of the CHEM 110 course. At the end of the semester, the project lead tabulated the number of surveys and reports submitted by each student and shared that information (student name and number of surveys and reports submitted) with each instructor to award extra credit points. Approximately 2 weeks after final grade submission for the course, instructors were asked to complete a post-survey after being provided with summary information on instructor and section-specific data. Data included mean hours per week their students spent (i) attending lecture, (ii) on additional study for the course, and (iii) devoted to the course overall. For comparison, instructors were provided with course-wide means. Prior to carrying out this project, the protocol was submitted to our Institutional Review Board (IRB) and, through an expedited review process, was approved as presenting minimal risk to human subjects (Protocol #: 2101212046).

### Analysis

The median (*M*_*d*_) was used to compare the central tendency of discrete data (i.e., number of metacognitive reports submitted by students) to the CHEM 110 letter grade earned. Categorical CHEM 110 letter grades earned were converted to numerical values on a standard four-point GPA scale (e.g., A = 4, B = 3, C = 2, D = 1, F/FSA = 0). Means and standard deviations were used for parametric two-sample independent *t* test comparisons between CHEM 110 course GPAs (reporting vs non-reporting student groups) and to check for equivalency of groups in terms of college entry indicators with effect sizes measured by eta-squared (η^2^). Effect size interpretations were informed by the small, medium, large scales established by Cohen as outlined by Privitera.^37^ Shapiro–Wilk tests indicated that course GPA and college entry indicator data were not normally distributed necessitating the use of the non-parametric Mann–Whitney U-test within DATAtab with the effect size (*r*) measured from the *z*-test statistic.

Chi-squared tests for independence between reporting and course success (number of ABC letter grades as proxy for course success) and reporting and gender were carried out with effect sizes measured by Cramer’s V. In a follow-up analysis, CHEM 110 final course GPAs were separated by gender with t- and U-test comparisons of mean course GPA for gendered categories of reporters and non-reporters.

The project lead, an experienced researcher and instructor, coded the post-survey student response data using inductive (data-driven) thematic coding.^38,39^ The iterative, reflective coding process included reading through student responses prior to establishing initial themes derived from the data, preliminarily coding a sampling of data to inform the creation of categories and subcategories, recategorization of themes, and/or combination of categories, and coding of all data followed by analysis (e.g., response frequencies and percentages). For consistency, a second coder checked a 10% sample of the student response data with resultant interrater reliability of 95%.

## Results and Discussion

### Characteristics of Metacognitive Reporters

To answer the first research question (RQ 1), reporting and non-reporting student data was compared. A total of 1,513 weekly reports were received over the 14 weeks of reporting (*M* = 108 reports per week). These reports were submitted by 300 distinct students (47.8% of students enrolled) with a median of 3 reports submitted per student and a range from 1 to 14. The number of reports submitted was related to the final CHEM 110 letter grade earned by the reporting students, as shown in Table 1. Students who received final letter grades of “A” in the course submitted more reports (*M*_*d*_ = 7, *n* = 88) than students who received final letter grades of “F” (earned or unearned) in the course (*M*_*d*_ = 1.5, *n* = 6).

**Table 1:** Median Number of Weekly Metacognitive Reports for Reporting Students (*n* = 300) by Final CHEM 110 Letter Grade Earned

Final Letter Grade	No. of Reporting Students	Median No. of Reports Submitted
A	88	7
B	108	4
C	49	3
D	11	3
F/FSA^a^	6	1.5
W^b^	38	2
All	300	3

a FSA is an unearned F for students who stopped attending the course.

b W is the grade for students who withdrew from the course at any time between the 2nd and 13th week of the semester.

In general, reporting students (*n* = 262) were more successful in the CHEM 110 course than non-reporting students (*n* = 269) as shown in Table 2. Reporting students finished the semester with a final course grade point average (GPA) 0.64 points higher than non-reporting students (0.69 points higher with inclusion of withdraws). There was a statistically significant difference between the course GPAs of reporting students (*M* = 3.00 GPA) versus non-reporting students (*M* = 2.36 GPA) indicating that reporting may have had a positive effect on earned GPA, *t*_529_ = 6.6652, *p* < 0.001 (η^2^ = 0.077). Non-normality of course GPA data (based on Shapiro–Wilk) prompted non-parametric re-evaluation of course GPA data with similar statistical significance of the results, *U* = 24816, *n*_1_ = 262, *n*_2_ = 269, *p* < 0.001 (*r* = 0.27).

**Table 2:** Average GPA and CHEM 110 Success (%ABC Letter Grades Earned) of Reporting Students versus Non-Reporting Students

CHEM 110 Student Population	No. of Students	Mean No. Grade Point Average (GPA)^a^	No. of ABC Letter Grades (%)
Reporting	262^b^	3.00 ± 0.95	245 (93.5%)
Non-Reporting	269^c^	2.36 ± 1.22	210 (78.1%)

a Data reported as mean ± standard deviation.

b Excludes 38 reporting students who withdrew from the course.

c Excludes 59 students who withdrew from the course.

In addition, a chi-square test for independence showed a significant relationship between reporting and course success (using number of A, B, or C letter grade attainment as a proxy for course success), *X*^2^(1, *n* = 531) = 25.815, *p* < 0.001, *V* = 0.22 (small to medium effect size, withdraws excluded). It could be argued that the 0.51 mean point “extra credit” increase to the final numerical grade was responsible for the success of reporting relative to non-reporting students. According to one instructor, who taught 33% of the CHEM 110 students and with more surveys and reports completed per student (*M*_*d*_ = 5, *M* = 6.6) than the average, the 0.62 mean point “extra credit” increase in final numerical grade for this instructor’s students moved only one student letter grade (from D to C) which would minimally affect the chi-squared analysis.

The two groups, reporting versus non-reporting students, were relatively equivalent based on both parametric and non-parametric comparisons of their college entry indicators. Mean and median verbal and combined SAT scores, English, science, reading, and combined ACT scores, and math placement exam scores were not significantly different between the two groups. Mean and median math SAT scores were significantly lower for reporting students than for non-reporting students by 11.03 and 10 points, respectively, but with small effect sizes (*t*_474_ = −2.859, *p* = 0.005 (η^2^ = 0.02); *U* = 244000.5, *n*_1_ = 228, *n*_2_ = 248, *p* = 0.01 (*r* = 0.12)). Math ACT scores were significantly lower for reporting students than for non-reporting students by 0.70 points in the parametric *t* test (*t*_247_ = −1.969, *p* = 0.049 (η^2^ = 0.02)) but were not significantly different in the non-parametric U-test. As a result, reporting students may have been slightly less prepared in math than non-reporting students. In contrast, reporting students had significantly higher mean and median high school GPAs than non-reporting students (mean: 3.92 vs 3.69; median: 3.96 vs 3.71) but once again with small effect sizes (*t*_612_ = 6.530, *p* < 0.001 (η^2^ = 0.07); *U* = 33389.5, *n*_1_ = 295, *n*_2_ = 319, *p* < 0.001 (*r* = 0.25)). However, there may be no practical significance to this GPA difference because our institution accepts unweighted and weighted high school GPAs and does not recalculate weighted GPAs according to a 4.0 scale.^40^ Thus, the CHEM 110 course success of reporting students relative to non-reporting students cannot be attributed to lack of equivalency in the two groups’ college entry indicators.

In addition, a chi-square test for independence on the gender of reporting versus non-reporting students indicated that significantly more females engaged in reporting than males with 64.0% (192, *n* = 300) and 45.1% (148, *n* = 328) of female students making up the reporting and non-reporting groups, respectively, *X*^2^(1, *N* = 628) = 22.490, *p* < 0.001, *V* = 0.19; whereas females constituted 54.1% (340, *N* = 628) of the population. The higher uptake of extra credit opportunities by female students is supported by the research of Harrison et al.^41^ Interestingly, separating the CHEM 110 final course grade data by gender and comparing the course GPAs showed a disparity between the genders. For both female and male students there were significant positive differences (on both parametric and non-parametric tests) in CHEM 110 course GPA between reporting and non-reporting students. The mean course GPAs of reporting female students (*M* = 2.59) were significantly higher than those of non-reporting female students (*M* = 2.10) by +0.49 (*t*_339_ = −3.19, *p* = 0.002 (η^2^ = 0.03); *U* = 11442.5, *n*_1_ = 148, *n*_2_ = 192, *p* = 0.002 (*r* = 0.17)). The mean course GPAs of reporting male students (*M* = 2.66) were significantly higher than non-reporting male students (*M* = 1.81) by +0.85 (*t*_287_ = −5.31, *p* < 0.001 (η^2^ = 0.09); *U* = 6271, *n*_1_ = 180, *n*_2_ = 108, *p* = 0.001 (*r* = 0.31)). However, higher CHEM 110 course GPAs were earned by males in the reporting group (female, *M* = 2.59; male, *M* = 2.66) but females in the non-reporting group (female, *M* = 2.10; male, *M* = 1.81), though these differences were not significant.

Overall and like the research of Casselman and Atwood^13^ and Hawker et al.,^14^ the data indicate that engaging in weekly metacognitive reporting may be associated with a greater proportion of students succeeding in the CHEM 110 course, although the effect size is small with reporting accounting for 5–8% of the total variance in course success. Though a positive correlation between weekly metacognitive reporting and course success exists, the causative direction (i.e., are successful students just more likely to submit more metacognitive reports) is not addressed by the data. In terms of characteristics, entry level indicators were roughly the same for students populating both groups, but in line with previous research,^41^ female students tended to engage in reporting at a higher rate than male students.

### Post-Survey Responses

#### Student Voiced Effects of Metacognitive Reporting

The second research question (RQ 2) was answered by qualitative analysis of student post-survey data. Of the 123 students who completed the post-survey, 117 provided a response to the open-ended question pertaining to student perceived effects from reporting. The results of the inductive thematic analysis are displayed in Table 3. By far, the largest category of response (72%, *n* = 84) was that students made modifications to their study methods or habits and attributed this to reporting. As the project intended, students were using the reporting as a metacognitive tool to hone in on challenging topics (39%, *n* = 46), reflect on study habits (37%, *n* = 43), and increase their study time (9%, *n* = 10). Likewise, students recognized that their instructors were reading the reports and responding to them (20%, *n* = 23) and that the reports promoted a good rapport with students (9%, *n* = 11).

**Table 3:** Frequencies and Percentages for Each Response Category of Student-Voiced Effects from Weekly Metacognitive Reporting (*n* = 117 Respondents)

Category	Response Frequency, *n* (%)	Subcategory Descriptor (*n*/%)^a^	Sample Student Statements
Students Made Modifications to Their Study Methods or Habits	84 (72%)	-helped identify or target study of challenging/hard topics (46/39%)	“...made me think about what topics I needed to work on. If I said that I didn’t feel comfortable about a subject, then that gave me the incentive to study for it before the next assessment.”
		-encouraged re-evaluation/reflection of study methods or habits (preparation, amount, approach, accountability) (43/37%)	*“It made me think about what kind of*habits I should think about, and perhaps what I should be doing to get more out of college. I did end up studying a bit more than I normally would have, though.”
		-indicated a need for more study time as observed from weekly time (hours) spent on class (lecture and additional study) (10/9%)	*“I think it helped me realize my study habits and improve them by seeing how many hours*I was studying during the week.”
		-had no effect on study methods or habits (8/7%)	
		-helped to view the course holistically (2/2%)	
Students Perceived That Instructors Responded to Reports	23 (20%)	-reviewed/addressed difficult concepts as informed by reports (12/10%)	*“It allowed the instructor to review concepts that we understood the least.”*
		-improved/tailored class experience or environment to students’ needs (12/10%)	*“My professor especially took the classes suggestions into consideration and implemented tools to help us during class.”*
Students Perceived Positive Impacts on Grades/Performance	12 (10%)	NA	*“It really helped on exams.”*
Students Perceived Improved Student-Instructor Interaction	11 (9%)	NA	“...[my instructor] understood what [instructor] needed to do to help the class understand the material better.”
Reporting Helped Non-Reporting Students	3 (3%)	NA	“...other students did the same [suggested topics for review] and some of the topics they needed to go over were ones I also wanted to review I had just forgotten about them...”

a Many student responses could be coded into 2 or more categories or subcategories. Thus, total category response frequency is larger than 117.

In accord with previous research,^29^ online weekly metacognitive reporting increased student-instructor interactions as evidenced by students’ post-survey statements. Students explicitly mentioned that instructors were (i) reading and responding to their recommendations, (ii) reviewing and addressing difficult concepts as informed by their reports, and (iii) tailoring the class experience or environment to students’ needs. In addition, one student expanded on two important aspects of the reporting mechanism: that it (i) provided anonymity and a space for less confident students to make recommendations and (ii) gave students a voice.

*“I think doing these reports turned out to be very helpful because they make our professor aware of questions and concerns we have while still maintaining our anonymity. Those with anxiety or difficulty asking questions in class or outside of class are given a voice.”*

#### Student Voiced Metacognitive Advice for Future Students

Qualitative analysis of student responses to a second post-survey question was used to answer the third research question (RQ 3). Of the 123 students who completed the post-survey, 120 provided a response to the question pertaining to course success and advice for future students. The results of inductive thematic coding are displayed in Table 4.

**Table 4:** Frequencies and Percentages for Each Response Category of Student-Voiced Advice for CHEM 110 Course Success (*n* = 120 Respondents)

Category	Response Frequency, *n* (%)	Subcategory Descriptor (*n*/%)^a^
Students Discussed:		
Engaging in Out-of-Class Practice/Study Activities	>58 (48%)	-complete/review online homework (36/30%)
		-review in-class problems (26/22%)
		-review past quizzes/exams (11/9%)
		-review lecture notes (11/9%)
		-review all material (1/1%)
Improving Study Habits	55 (46%)	-spread out study/review for long-term learning (21/18%)
		-put more effort into studying (21/18%)
		-stay on track/focused, caught up, do not procrastinate (14/12%)
		-memorize specific things (ion charges, rules) (8/7%)
		-do not try to memorize everything (2/2%)
Seeking Understanding from Course Instructor	39 (32%)	-ask questions (24/20%)
		-attend office hours or schedule meeting (11/9%)
		-follow instructions, methods, or guidance (6/5%)
		-ask for help (2/2%)
The Importance of In-Class Activities	35 (29%)	-attend class (19/16%)
		-pay attention and/or participate in class (15/12%)
		-take notes (9/8%)
Seeking Understanding from Others	15 (12%)	-attend tutoring (7/6%)
		-study with group or friends (7/6%)
		-attend study hours (1/1%)
Engaging in Other Out-of-Class Practice/Study Activities	9 (8%)	-prepare study guides (3/2%)
		-watch YouTube videos (3/2%)
		-make notecards/flashcards (2/2%)
		-complete extra credit (1/1%)
		-complete learning checks (1/1%)
Not Allowing Low Score(s) Early in the Semester Define Continued Performance	3 (2%)	NA

a Many student responses could be coded into 2 or more categories or subcategories. Thus, total category response frequency is larger than 120.

Student-voiced recommendations were aligned with statements outlined by McGuire and McGuire’s Learning Strategies Inventory (p 179).^35^ Students indicated or recommended reviewing lecture notes (9%, *n* = 11) and/or in-class problems (22%, *n* = 26), completing/reviewing homework (30%, *n* = 36), and reviewing quizzes or exams for errors (9%, *n* = 11). Out-of-class, students reiterated the importance of attending office hours (9%, *n* = 11) or participating in study groups (6%, *n* = 7). Further, students recognized the importance of studying progressively and at frequent times throughout the week (18%, *n* = 21) and many advocated for more effort toward studying (18%, *n* = 21). A few students (2%, *n* = 3) also recognized that they could do well in the course, despite a few low quiz grades. In fact, many student recommendations promoted study skills, such as attending class (16%, *n* = 19), paying attention and participating in lecture (12%, *n* = 15), and taking notes (8%, *n* = 9), that Atieh et al. found were aligned with “deep learning” and success in general chemistry.^42^ Notably, only three students advised watching YouTube videos to improve their success in the CHEM 110 course. We were pleased with this result as recent literature indicates that watching YouTube videos that are not instructor-generated or recommended may be detrimental to at-risk students and correspond to students with high levels of surface-skill learning (i.e., passive learning focused on memorization and an inability to move from rote to meaningful learning).^42^ Students were forthright in their advice, as exemplified by the following excerpted student comments:

*“STUDY STUDY STUDY. Showing up to class is crucial.”*

*“It was very important for me to realize that a couple of poor grades won’t matter in the long run if I was able to get other grades up.”*

*“...ask questions when they have them because it is better to ask when you are confused in the moment...”*

*“Really work on long-term memory for things. A lot of the time I would memorize something for the quiz or test and then forget so now I have to relearn all those things.”*

*“GO TO TUTORING if you need the help, this makes you a proactive student and will help your grade. Going to tutoring does not mean you’re dumb it means you’re smarter than your peers for seeking out help.”*

*“Stay on top of their work and don’t slack off. This is the only way that you will succeed...always pay attention in class so they can have a good understanding of everything going on at all times. This is what I wish I was told by another student...”*

#### Instructor Use of Metacognitive Reports

Responses to the instructor post-survey were used to answer the fourth research question (RQ 4). Four of the five CHEM 110 instructors responded to the post-survey. All four were (i) experienced instructors and (ii) in positions dedicated to chemistry teaching at the undergraduate level. All four instructors agreed that excellent instruction is important for their positions. Instructors self-reported reading over an average of 87% of the 14 weekly embedded reports (*s* = 19%, range 60% to 100%). Each Sunday morning, reports for the week were sorted and separated by section and instructor and were anonymized. Instructors received section specific student feedback by 1 PM on Sunday with the goal that weekly student feedback would be timely enough to inform their teaching on Monday. All of the instructors indicated that the reports were timely enough to inform their teaching. As shown below, one instructor mentioned the small amount of time needed to review reports, and another mentioned that the reports gave them an overall impression of class climate.

*“They were normally available on Sunday and I only needed about 20 mins to review the reports and make the decision to change my plans.”*

*“It was useful to review the “sense of the class” shortly before lecture. We all continuously attempt to read our students facial expressions and body language - but not all students are open with non-verbal expressions.”*

Instructors self-reported making an average of 4.5 instructional adjustments based on student feedback from the reports. Reported adjustments included:

- providing more in-class practice problems on difficult concepts
- providing a video on nomenclature to supplement in-class teaching
- additional review of significant figures
- providing answer keys to extra practice problems
- modifying the lighting and sound in the classroom (e.g., microphone and screen projection)
- addressing comments on the class pace and encouraging students to use posted slide outlines for notetaking (but notably not reducing the amount of material covered)
- implementing “problem-solving/review days” before each major assessment
- a halt to presentation of new material and instead revisiting an older, critical topic
- reworking material into reviews or new lectures to revisit materials that students did not understand previously
- referring students to instructor/institutional lesson videos and problem-solving videos for clarification or review

Beyond instructional adjustments, instructors indicated that the reports provided them with students’ mindset toward the content and learning environment. As one instructor wrote, the weekly reports provided “*A general idea of what students thought of the material and how much students were working beyond the classroom sessions*”. Perhaps, more importantly, instructors recognized that weekly reporting provided their students with an anonymous, student voice to inform the instructor’s ongoing classroom actions as illustrated by the following statements:

*“By answering questions and addressing ideas from the surveys in class my students felt heard. They knew I was reading them and taking time to listen and work to answer their concerns about the material.”*

*“Students [were] very very appreciative that they were allowed this means of anonymous feedback, especially on the days that I explicitly told them that I was responding directly to their comments.”*

### Limitations

Limitations include the following:

- Beyond our institution, results may not be generalizable. Project results are specific to our institution.
- Although all students were invited to participate, students self-selected to participate in one or more of the reporting opportunities. This self-selection could be a confounding variable.
- Over-reporting or under-reporting of numbers—student estimates of hours spent on additional class study and instructor estimates of percentage of reports read—is a distinct possibility as weekly reporting was removed in time from activities.
- Post-survey results are more representative of the attitudes of students who had success in the course. The student post-survey was unavailable to students who withdrew from the course. In addition, students who were expecting to fail the course had minimal incentive to complete the post-survey.
- Students in different sections experienced slight alterations in classroom environment because of the variation in meeting days and times and in assigned instructor. Although the instructors ran the course as a team and the course pedagogy was coordinated, instructors are individuals and have different personalities and unique approaches to explaining content.

Despite these limitations, this project involved a large population of students who submitted robust reports that provided important insights into students’ lived experiences in a large enrollment introductory chemistry course. In fall 2023, we duplicated this study in a general chemistry 1 course with comparable findings. In addition, we are working with instructors at our institution and beyond to implement similar projects in their STEM courses. To limit the impact of self-selection, we are encouraging frequent metacognitive reporting as an integral part of the course instead of as extra credit assignments.

## Conclusions

Analysis showed that a modest amount of extra credit incentivized students to submit 1,513 weekly metacognitive reports over the course of 14 weeks with a median of 3 reports submitted per reporting student. As intended, weekly metacognitive reporting provided introductory chemistry students with an opportunity to practice metacognition, despite the absence of explicit training in metacognitive strategies. The metacognitive reporters earned significantly higher final course grades than non-reporters (0.64 GPA points higher and 15.4% more ABC letter grades) but with small effect sizes. Although the data do not explain the directionality of this effect, our limited findings are in accord with previous research that providing opportunities for students to implicitly practice metacognition improves their success in chemistry.^13,14^

Statistical analysis of college entry indicators suggests that the two student groups, reporters versus non-reporters, were not obviously different as to their readiness for college coursework. Interestingly, the reporting group contained a significantly higher percentage of female students than the non-reporting group in line with previous research.^41^ Both female and male reporting students received higher course grades than non-reporting students, but again, the statistical effect sizes were small. However, like gendered studies of online homework use in general chemistry,^43^ male students would seem to have benefitted more than female students.

On the post-survey, reporting students indicated being prompted to use metacognitive strategies of identifying and targeting study toward challenging topics, reflecting on and changing study habits to improve learning, and increasing their study time for the course. One student captured the intended metacognitive impact of reporting with the following statement:

*“It affected me this semester because it allowed me to analyze myself in ways that I didn’t take to [sic] serious in prior years of schooling. This ultimately allowed me to do better in the course and apply myself way more. I realized what I was doing wasn’t working and that I needed to change my habits. Now, I feel that I am more prepared for future classes.”*

Students’ advice to future students for success in the Introduction to Chemistry course was metacognitively sound (e.g., attend lecture, participate and pay attention, take notes, study progressively, complete the homework) and aligned with recommendations for “deep learning”.^42^

Weekly metacognitive reporting and student observations of how instructors responded to reports not only improved student-instructor interactions but contributed to student’s view of instructors as beneficent. This is aligned with previous research by Christe^25^ and others^26−30^ that support instructor accountability to students via positive student-instructor interactions and high levels of care and compassion. As indicated by Ramsden, with this project the instructors were prompted to “see learning through the learner’s eyes” via student-voiced, weekly metacognitive reporting.^44^

Instructors can use these findings to inform their pedagogy, especially for teaching chemistry to students at the introductory levels. It is recommended that instructors create frequent, low-stakes assignments built into the course structure to support their students’ implicit use of metacognition with the broad goal of growing metacognitive strategies over time. As higher achieving students tend to complete “extra credit” more often than lower-achieving students,^41,45,46^ metacognitive assignments that are required of all students (i.e., in place of self-selection for “extra credit”) could partially mitigate the likelihood that only higher achieving students would benefit. In addition, a dual strategy of explicit teaching about metacognition parallel with implicit practice of metacognitive strategies can be explored. Lastly, instructors should be encouraged to elevate student voices and be proactive in responding to anonymous student input through teaching modifications. This will establish a more positive relationship between students and instructors and improve students’ view of the instructor as beneficent.

Future research to definitively answer the question of whether metacognitive reporting leads to improved success in chemistry coursework is envisioned. For instance, metacognitive reporting could be implemented as a required course component. After dividing the students in the course into two equivalent groups, the first group could be required to submit metacognitive reports for the first and third exams and the second group for the second and fourth exams. In this way, both student groups would receive the same benefit, whereas exam scores (reporting vs non-reporting groups) could be compared for each exam to assess the effects of metacognitive reporting.

Overall, this project’s results add to our understanding of students’ metacognition and how instructors can pro-actively support students in developing their metacognitive abilities to facilitate chemistry learning. Further, discussion of projects informed by “student voice” (or projects informed by students instead of done to students) is limited in the literature. This project expands the conversation on how “student voice” can be used to positively impact the student-instructor relationship for large enrollment chemistry courses.

## Supporting Information Available

The four surveys that were used (you are welcome to customize these surveys for your own study): Embedded Student Weekly Metacognitive Report (for Introductory Chemistry/CHEM 110), Student Pre-Survey Instrument, Student Post-Survey Instrument, and Instructor Embedded Student Post-Survey Instrument (PDF)
