# Supplementary material for: Promotion of Student Success and Positive Chemistry Course Perception through Frequent Metacognitive Reporting
Source: J Chem Educ. 2025 Jan 1;102(1):102–11. doi: 10.1021/acs.jchemed.4c00578 (PMC11736795; doi:10.1021/acs.jchemed.4c00578)
Supplement: Supplementary file 1 — ed4c00578_si_001.pdf [file ed4c00578_si_001.pdf]

Promotion of Student Success and Positive Chemistry Course Perception Through Frequent Metacognitive Reporting

Michelle Richards-Babb*, Carly Gordon, David Mersing, Trina Perrone, and Betsy Ratcliff

C. Eugene Bennett Department of Chemistry, West Virginia University, Morgantown, WV 26506, United States

Corresponding Author: *E-mail: Michelle.Richards-Babb@mail.wvu.edu

Embedded Student Weekly Report (Fall 2022)

Start of Block: Embedded Student Weekly Report

Fall 2022: **Embedded Student Weekly Reports** (for Chem 110) are due before 11:59 pm on Saturday of each academic week. Report (i) hours devoted to attending the lecture portion of your Chem 110 course and (ii) hours devoted to additional study for the lecture portion of your Chem 110 course. Reporting will be available continuously throughout the Fall 2022 semester and will be due by/before 11:59 pm each Saturday, including

Aug. 27; Sept. 3, 10, 17, & 24; Oct. 1, 8, 15, 22, & 29; Nov. 5, 12, & 19; and Dec 3

Hours devoted to attending lecture include attending regularly scheduled weekly in-person lecture class or class problem-solving sessions, viewing virtual/online synchronous or asynchronous class videos, and taking online or in-person exams and quizzes. Hours devoted to additional study include reviewing/rewriting class notes, completing assigned homework (graded or ungraded), studying for quizzes and exams, etc. Additional study typically occurs outside of the regularly scheduled lecture class.

Thus, each day and each week, keep track of hours devoted to attending and studying for your Chem 110 course and provide additional details as outlined below. Each week, your name will be stripped from the report and your report will be sent to course instructors who have agreed to receive these reports. Reports will serve to provide instructors with formative feedback from the perspective of their students (i.e., student voice). Questions or concerns?
Contact Michelle.Richards-Babb@mail.wvu.edu.

The report you are submitting is for the week of:

- ◯ Week 1: Aug 21-27
- ◯ Week 2: Aug 28-Sept 3
- ◯ Week 3: Sept 4-10
- ◯ Week 4: Sept 11-17
- ◯ Week 5: Sept 18-24
- ◯ Week 6: Sept 25-Oct 1
- ◯ Week 7: Oct 2-8
- ◯ Week 8: Oct 9-15
- ◯ Week 9: Oct 16-22
- ◯ Week 10: Oct 23-29
- ◯ Week 11: Oct 30-Nov 5
- ◯ Week 12: Nov 6-12
- ◯ Week 13: Nov 13-19
- ◯ Week 14: Nov 27-Dec 3

Enter your first name (and middle initial if you have one) and last name below. Needed to award credit/points for completing survey.

- ◯ First Name and middle initial ______________________________
- ◯ Last Name ______________________________

What is your complete institutional email address?

- ○ Email Address ____

What is the name of your Chem 110 course instructor?

- ○ Instructor A
- ○ Instructor B
- ○ Instructor C
- ○ Instructor D
- ○ Instructor E

What is the email address of your Chem 110 course instructor?

- ○ InstructorA@mail.wvu.edu
- ○ InstructorB@mix.wvu.edu
- ○ InstructorC@mail.wvu.edu
- ○ InstructorD@mail.wvu.edu
- ○ InstructorE@mail.wvu.edu

In which Chem 110 section are you enrolled? Needed to award credit/points for completing survey.

- 001 (MWF, 8:30 am)
- 002 (MWF, 2:30 pm)
- 003 (MWF, 10:30 am)
- 004 (MWF, 11:30 am)
- 005 (TR, 8:30 am)
- 006 (TR, 4:00 pm)
- 007 (TR, 2:30 pm)
- 008 (MWF, 3:30 pm)
- 009 (MWF, 4:30 pm)
- 010 (TR, 10:00 am)

Report your hours devoted to attending lecture for the Chem 110 course to the nearest quarter hour (e.g., 0.50, 1.25, 2.00, 3.75, etc.). Do NOT: include units, enter time in minutes or a combination of hours:minutes, or enter anything other than numbers and decimal marks.

Note: 50-minute lectures can be entered as 0.83. 75-minute lectures can be entered as 1.25.

Hours devoted to lecture include attending regularly scheduled weekly in-person lecture class or class problem-solving sessions, viewing virtual/online synchronous or asynchronous class videos, and taking online or in-person exams and quizzes.

	Hours devoted to attending lecture each day (e.g., 1.25)

	Sun	Mon	Tues	Wed	Thur	Fri	Sat
Past Week							

Please provide a detailed summary of your lecture attending activities during the past week. Provide more, rather than less, detail. In addition, address whether you found your time attending Chem 110 lecture useful for out-of-lecture additional study activities (e.g., Did the time spent doing in class lecture activities help you know how to approach the homework problems?).

____

____

____

____

____

Please report your hours devoted to additional study for the Chem 110 course to the nearest quarter hour (e.g., 0.50, 1.25, 2.00, 3.75, etc.). Do NOT: include units, enter time in minutes or a combination of hours:minutes, or enter anything other than numbers and decimal marks.

Hours devoted to additional study include reviewing/rewriting class notes, completing assigned homework (graded or ungraded), studying for quizzes and exams, etc. Additional study typically occurs outside of the regularly scheduled lecture class.

	Hours devoted to additional study each day (e.g., 1.25)

	Sun	Mon	Tues	Wed	Thur	Fri	Sat
Past Week							

Please provide a detailed summary of your additional study activities during the past week. Provide more, rather than less, detail.

____

____

____

____

____

In journal form, identify those Chem 110 course content topics that you feel you have mastered during the past week. **Mastery** implies that you feel you could "teach" the topics to a fellow student.

What about these topics (inherent in the nature of the topics, how these topics were taught, or your past experience) helped you to master these topics?

Use complete sentences and provide more, rather than less, detail.

____

____

____

____

____

In journal form, identify those Chem 110 course content topics that you feel comfortable with from the past week. **Comfortable** implies that you feel you understand the topics but are not yet ready to "teach" these topics to a fellow student.

What about these topics is still fuzzy (in terms of your understanding, familiarity, applications) and is impeding your ability to master these topics? What will you do to improve your understanding of these topics?

Use complete sentences and provide more, rather than less, detail.

In journal form, identify those Chem 110 course content topics that you find totally confusing from the past week. **Total confusion** implies that you feel you do not understand the topics and would have trouble answering questions on these topics.

What about these topics is confusing and why? What will you do to improve your understanding of these topics?

Use complete sentences and provide more, rather than less, detail.

Display This Question:

If The report you are submitting is for the week of: = Week 1: Aug 21-27

Or The report you are submitting is for the week of: = Week 14: Nov 27-Dec 3

Or The report you are submitting is for the week of: = Week 7: Oct 2-8

Pertaining to the Chem 110 course:	Not at all true (1)	Untrue (2)	Neither true or untrue (3)	True (4)	Very True (5)
I feel confident in my ability to learn the content for this course.	○	○	○	○	○
I am capable of learning the material/content in this course.	○	○	○	○	○
I am able to achieve my goals for this course.	○	○	○	○	○
I feel able to meet the challenges of performing well in this course.	○	○	○	○	○
I feel comfortable communicating (in-person, email, phone, etc.) with the instructor of this course.	○	○	○	○	○
I feel comfortable communicating (in-person, email, phone, etc.) with fellow students in this course.	○	○	○	○	○
I know what I need to do to improve my understanding of the content for this course.	○	○	○	○	○

I am doing all that I can to improve my understanding of the content for this course.	○	○	○	○	○

If you could tell your Chem 110 course instructor of one thing that helped you learn this past week, what would it be?

____

____

____

____

____

If you could tell your Chem 110 course instructor about one thing that could improve your learning this past week, what would it be?

____

____

____

____

____

End of Block: Embedded Student Weekly Report

Chem 110 Learning Strategies Evaluation: Pre-survey (Fall 2022)

Start of Block: Default Question Block

Please evaluate each of the following statements using the scale shown below each statement. Your name is needed to match your pre-semester and post-semester responses and inform your instructor of survey completion for potential course credit. A third-party person, not your instructor, will match pre-and post-semester responses and analyze all data. Your instructor will never know your specific response to any of the survey questions.

Note that you do not have to answer every question.
Questions? Contact Michelle Richards-Babb (mrichar2@wvu.edu)

X→

Please mark the response that best describes your beliefs **at this time** as pertains to your learning in science and mathematics and/or STEM courses.

	Strongly Disagree (1)	Disagree (2)	Neutral (Neither Agree nor Disagree) (3)	Agree (4)	Strongly Agree (5)
If I fail or perform poorly on a quiz or an exam, my instructor is more to blame than I.	○	○	○	○	○
I am more responsible than my instructor for ensuring that I learn and understand the content in science, mathematics and/or STEM courses.	○	○	○	○	○
I am very confident in my ability to use formulas and equations.	○	○	○	○	○
I am very confident in doing homework problems well.	○	○	○	○	○
I am very confident in asking questions during and outside of class (in-person and/or virtual).	○	○	○	○	○
I am very confident in understanding concepts from course textbooks.	○	○	○	○	○

I am very confident about getting good grades in science, mathematics and/or STEM courses.	○	○	○	○	○
I am very confident in understanding abstract science, mathematics, and STEM concepts.	○	○	○	○	○
My understanding and grades in science, mathematics and/or STEM courses are determined more by how “smart” I am than by how much homework I complete.	○	○	○	○	○

Think about the **LAST** science, mathematics, and/or STEM course you took. Base your responses to the statements below on your study habits for that **previous course**.

	Strongly Disagree (1)	Disagree (2)	Neither Agree nor Disagree (3)	Agree (4)	Strongly Agree (5)
I always previewed the material that was to be discussed before I attended class.	○	○	○	○	○
I went over my lecture notes as soon as possible after lecture to rework them and note problem areas.	○	○	○	○	○
I tried to do my homework without using example problems as a guide or copying answers from my class notes or textbook.	○	○	○	○	○
I went to office hours or tutoring regularly to discuss problems or questions on the homework.	○	○	○	○	○
I reworked all of the homework problems and questions before the test or quiz.	○	○	○	○	○
I spent some time studying for the class at least five days per week (outside of class time).	○	○	○	○	○

I made mnemonics for myself to help me remember facts and equations.	○	○	○	○	○
I made diagrams or drew mental pictures of the concepts discussed in class.	○	○	○	○	○
I participated in a study group where we did homework and quizzed ourselves on the material.	○	○	○	○	○
I reworked all of the quiz and test items I had missed before the next class session.	○	○	○	○	○
I realized that I could still do well in the class even if I had done poorly on initial quizzes and tests.	○	○	○	○	○

The following questions relate to your reasons for participating actively in your science, mathematics, and/or STEM courses. Different people have different reasons for their participation. We want to know how true each of these reasons is for you.

I will participate actively in my science, mathematics, and/or STEM courses:

	Not at all True (1)	Untrue (2)	Neutral (Neither True or Untrue) (3)	True (4)	Very True (5)
because I feel like it's a good way to improve my understanding of the material.	○	○	○	○	○
because others might think badly of me if I didn't.	○	○	○	○	○
because I would feel proud of myself if I did well in the course.	○	○	○	○	○
because a solid understanding of science, mathematics and/or STEM is important to my intellectual growth.	○	○	○	○	○

I am likely to follow my instructor's suggestions for studying for my science, mathematics, and/or STEM courses:

	Not at all True (1)	Untrue (2)	Neutral (Neither True or Untrue) (3)	True (4)	Very True (5)
because I would get a bad grade if I didn't do what they suggest.	○	○	○	○	○
because I am worried that I am not going to perform well in these courses.	○	○	○	○	○
because its easier to follow their suggestions than coming up with my own study strategies.	○	○	○	○	○
because they seem to have insight about how best to learn the material.	○	○	○	○	○

The reason that I will work to expand my knowledge of science, mathematics, and/or STEM is:

	Not at all True (1)	Untrue (2)	Neutral (Neither True or Untrue) (3)	True (4)	Very True (5)
because it’s interesting to learn more about science, mathematics, and/or STEM.	○	○	○	○	○
because it’s a challenge to really understand how to solve science, mathematics, and/or STEM problems.	○	○	○	○	○
because a good grade in science, mathematics, and/or STEM courses will look positive on my record.	○	○	○	○	○
because I want others to see that I am intelligent.	○	○	○	○	○

In which Chem 110 section (instructor) combination are you enrolled for Fall 2022? Needed to award credit/points for completing survey.

- ○ Section 001 (Instructor X)
- ○ Section 002 (Instructor X)
- ○ Section 003 (Instructor X)
- ○ Section 004 (Instructor X)
- ○ Section 005 (Instructor X)
- ○ Section 006 (Instructor X)
- ○ Section 007 (Instructor X)
- ○ Section 008 (Instructor X)
- ○ Section 009 (Instructor X)
- ○ Section 010 (Instructor X)

What is your first name and middle initial (if you have one)? Needed to award credit/points for completing survey.

____

What is your last name? Needed to award credit/points for completing survey.

____

End of Block: Default Question Block

Chem 110 Learning Strategies Evaluation: Post-survey (Fall 2022)

Start of Block: Default Question Block

Please evaluate each of the following statements using the scale shown below each statement. Your name is needed to match your pre-semester and post-semester responses and inform your instructor of survey completion for potential course credit. A third-party person, not your instructor, will match pre-and post-semester responses and analyze all data. Your instructor will never know your specific response to any of the survey questions.

Note that you do not have to answer every question.

Questions? Contact Michelle Richards-Babb (mrichar2@wvu.edu)

Please mark the response that best describes your beliefs at this time as pertains to your learning in science and mathematics and/or STEM courses.

	Strongly Disagree (1)	Disagree (2)	Neutral (Neither Agree nor Disagree) (3)	Agree (4)	Strongly Agree (5)
If I fail or perform poorly on a quiz or an exam, my instructor is more to blame than I.	○	○	○	○	○
I am more responsible than my instructor for ensuring that I learn and understand the content in science, mathematics, and/or STEM courses.	○	○	○	○	○
I am very confident in my ability to use formulas and equations.	○	○	○	○	○
I am very confident in doing homework problems well.	○	○	○	○	○
I am very confident in asking questions during and outside of class (in-person and/or virtual).	○	○	○	○	○
I am very confident in understanding concepts from course textbooks.	○	○	○	○	○

I am very confident about getting good grades in science, mathematics and/or STEM courses.	○	○	○	○	○
I am very confident in understanding abstract science, mathematics and/or STEM concepts.	○	○	○	○	○
My understanding and grades in science, mathematics and/or STEM courses are determined more by how “smart” I am than by how much homework I complete.	○	○	○	○	○

Think about the science, mathematics, and STEM courses you took during the Fall 2022 semester. Base your responses to the statements below on your study habits for those courses.

	Strongly Disagree (1)	Disagree (2)	Neither Agree nor Disagree (3)	Agree (4)	Strongly Agree (5)
I always previewed the material that was to be discussed before I attended class.	○	○	○	○	○
I went over my lecture notes as soon as possible after lecture to rework them and note problem areas.	○	○	○	○	○
I tried to do my homework without using example problems as a guide or copying answers from my class notes or textbook.	○	○	○	○	○
I went to office hours or tutoring regularly to discuss problems or questions on the homework.	○	○	○	○	○
I reworked all of the homework problems and questions before the test or quiz.	○	○	○	○	○
I spent some time studying for the class at least five days per week (outside of class time).	○	○	○	○	○

I made mnemonics for myself to help me remember facts and equations.	○	○	○	○	○
I made diagrams or drew mental pictures of the concepts discussed in class.	○	○	○	○	○
I participated in a study group where we did homework and quizzed ourselves on the material.	○	○	○	○	○
I reworked all of the quiz and test items I had missed before the next class session.	○	○	○	○	○
I realized that I could still do well in the class even if I had done poorly on initial quizzes and tests.	○	○	○	○	○

The following questions relate to your reasons for participating actively in your science, mathematics and/or STEM courses. Different people have different reasons for their participation. We want to know how true each of these reasons is for you.

I participated actively in my science, mathematics and/or STEM courses during the Fall 2022 semester:

	Not at all True (1)	Untrue (2)	Neutral (Neither True or Untrue) (3)	True (4)	Very True (5)
because I feel like it's a good way to improve my understanding of the material.	○	○	○	○	○
because others might think badly of me if I didn't.	○	○	○	○	○
because I would feel proud of myself if I did well in the courses.	○	○	○	○	○
because a solid understanding of science, mathematics, and STEM is important to my intellectual growth.	○	○	○	○	○

I am likely to follow my instructor’s suggestions for studying for my science, mathematics and/or STEM courses:

	Not at all True (1)	Untrue (2)	Neutral (Neither True or Untrue) (3)	True (4)	Very True (5)
because I would get a bad grade if I didn't do what they suggest.	○	○	○	○	○
because I am worried that I am not going to perform well in these courses.	○	○	○	○	○
because its easier to follow their suggestions than coming up with my own study strategies.	○	○	○	○	○
because they seem to have insight about how best to learn the material.	○	○	○	○	○

The reason that I will work to expand my knowledge of science, mathematics and/or STEM is:

	Not at all True (1)	Untrue (2)	Neutral (Neither True or Untrue) (3)	True (4)	Very True (5)
because it's interesting to learn more about science, mathematics and/or STEM.	○	○	○	○	○
because it's a challenge to really understand how to solve science, mathematics and/or STEM problems.	○	○	○	○	○
because a good grade in science, mathematics and/or STEM courses will look positive on my record.	○	○	○	○	○
because I want others to see that I am intelligent.	○	○	○	○	○

How did participating in "embedded student" reporting for Chem 110 affect you this semester? Did you think about or do anything differently that you would attribute to the reporting? Did writing your reports affect how you studied for your Chem 110 or other STEM, math and/or science classes (i.e., your thinking or decision-making)? Please explain thoroughly.

____

____

____

____

If you were to advise a future student for success in this **Chem 110** course, what advice would you give? In hindsight, what did you do that was important to your success and what would you have done differently?

In which Chem 110 section (instructor) combination are you enrolled for Fall 2022? Needed to award credit/points for completing survey.

- ◯ Section 001 (Instructor X)
- ◯ Section 002 (Instructor X)
- ◯ Section 003 (Instructor X)
- ◯ Section 004 (Instructor X)
- ◯ Section 005 (Instructor X)
- ◯ Section 006 (Instructor X)
- ◯ Section 007 (Instructor X)
- ◯ Section 008 (Instructor X)
- ◯ Section 009 (Instructor X)
- ◯ Section 010 (Instructor X)

What is your first name and middle initial (if you have one)? Needed to award credit/points for completing survey.

____

What is your last name? Needed to award credit/points for completing survey.

____

Page Break

End of Block: Default Question Block

Chem 110 - Embedded Instructor Post-Survey (Fall 2022)

Start of Block: Chem 110 - Embedded Student Weekly Report

Fall 2022: Chem 110 - **Embedded Student Project Instructor Post-Survey** Dear Instructors: Thank you for agreeing to take part in the Embedded Student Project during Fall 2022. We would like to gather your impressions of the project and the reports to improve them for upcoming semesters.

Questions or concerns? Contact Michelle.Richards-Babb@mail.wvu.edu.

Approximately what percentage of the embedded students reports that you were sent were you able to read over?

	0 10 20 30 40 50 60 70 80 90 100
Percentage of Reports Read ()	

Were the reports timely enough to inform your teaching beginning on Monday? Why or why not? Please explain.

____

____

____

____

____

In general, what did the embedded student feedback reveal about your students' understanding of content from the previous week?

____

____

____

____

____

Approximately how many times did you use feedback from the embedded student reports to make instructional adjustments (i) to improve your effectiveness as an instructor and/or (ii) to improve student learning?

▼ None ... 8 or more

Page Break

What instructional adjustments did you make as a result of the embedded student feedback? List and discuss as many adjustments as you can remember.

____

____

____

____

____

Overall, how successful and/or effective were these adjustments?

▼ Extremely successful ... Not successful

Was the feedback from the embedded reports useful beyond informing instructional adjustments? If so, list and discuss other ways the feedback was useful.

____

____

____

____

____

You were sent an email documenting the average number of hours per week your embedded student(s) (i) spent attending/viewing lecture, (ii) completing additional study, and (iii) devoted to your course overall (lecture plus additional study). Were the average number of hours per week the student(s) devoted to class overall (lecture plus additional study) surprising?

▼ No, the hours were about what I expected. ... Yes, the hours were MORE than I expected.

Please explain your response to the previous question in regards to average number of hours per week overall devoted to your class.

____

____

____

____

____

Would you like to receive embedded student reports during the Spring 2023 semester?

▼ Yes ... Maybe

Explain your response to the previous question regarding your willingness to receive embedded student reports during the Spring 2023 semester.

____

____

____

____

____

Would you recommend the Embedded Student Project to other instructors?

▼ Yes ... Maybe

Explain your response to the previous question regarding your recommendation of the embedded student project to other instructors.

____

If you are including your involvement in the embedded student project in your annual promotion and tenure report, please explain what information you are including.

Is there any other information you would like to have that the students did not provide in the embedded reports? For instance, are there additional questions you would like to have included on the embedded report form?

Do you have any recommendations for improving the embedded student project? If so, please detail your recommendations below.

Indicate how strongly you agree or disagree with each statement below.

	1 (Strong Disagree)	2 (Disagree)	3 (Neither Agree or Disagree)	4 (Agree)	5 (Strongly Agree)
Providing excellent instruction is a strong expectation at my institution.	○	○	○	○	○
My job is simply to present information to the students.	○	○	○	○	○
Providing excellent instruction is a major component of my duties as a faculty member.	○	○	○	○	○
Providing excellent instruction is crucial to my success in my department.	○	○	○	○	○
I received formal training in effective teaching methods while in graduate school.	○	○	○	○	○
Research productivity is more important than instructional innovation in my professional success.	○	○	○	○	○

It is my responsibility to create a classroom environment that requires students to think critically.	○	○	○	○	○
I have received support from colleagues in my attempts to improve my teaching.	○	○	○	○	○
It is important to experiment with (try out) new instructional techniques in the classroom.	○	○	○	○	○

For which course and number did you receive embedded reports? Enter as, e.g., Chem 110.

____

What was the modality for your section of this course during Fall 2022? Consider only the actual lecture part of the course.

○ Totally virtual or online (no in-person component)

○ Hybrid or HyFlex (part online and part in-person, which means that students can participate online or in-person, at their discretion)

○ Face-to-face (totally in-person, no online component)

What is your current rank? I am a/an:

▼ Graduate Teaching Assistant ... Other

Do you have tenure?

▼ Yes ... My position is not a tenure-track position.

How long have you been teaching at the college level:

▼ 0-4 years ... 20+ years

End of Block: Chem 110 - Embedded Student Weekly Report
